# Optimization of a real municipal sewage treatment plant using CRFSMA algorithm and a mathematical model

**DOI:** 10.1016/j.heliyon.2024.e34785

**Published:** 2024-07-21

**Authors:** Chunchang Lu, Ying Chen, Behrooz Eskandarpour, Khalid A. Alnowibet

**Affiliations:** a Chongqing Vocational Institute of Engineering, Chongqing, 402260, China; b Chongqing Academy of governance, Jiulongpo, Chongqing, 400039, China; c Department of Industrial Engineering, Ankara Yıldırım Beyazıt University (AYBU), Ankara 06010, Turkey; d Statistics and Operations Research Department, College of Science, King Saud University, Riyadh, 11451, Kingdom of Saudi Arabia

**Keywords:** Biological wastewater treatment, Multi-criteria optimization, CRFSMA, Energy consumption, Wastewater quality

## Abstract

This study presents the development, calibration, and validation of a mathematical model tailored for biological wastewater treatment at an actual urban sanitation facility. Utilizing multi-criteria optimization techniques, the research identified the most effective MCO algorithm by assessing Pareto optimal solutions. The model incorporated three primary performance measures energy consumption, overall volume, mean quality of effluent, and optimized 12 process parameters. Three algorithms, CRFSMA, particle swarm algorithm, and adaptive non-dominated sorting genetic algorithm III, were rigorously tested using MATLAB. The CRFSMA method emerged as superior, achieving enhanced Pareto optimal solutions for three-dimensional optimization. Quantitative improvements were observed with a 14.8 % increase in wastewater quality and reductions in total nitrogen (TN), chemical oxygen demand (COD), total phosphorus (TP), and ammonium nitrogen (${NH}_{4}^{+}$-N) concentrations by 0.95, 2.38, 0.04, and 0.14 mg/L, respectively. Additionally, the total cost index and overall volume were decreased, contributing to an 18.27 % reduction in overall volume and an 18.83 % decrease in energy utilization. The adapted anaerobic-anoxic-Oxic (A^2^O) framework, based on real-world wastewater treatment plants, demonstrated compatibility with observed effluent variables, signifying the potential for energy savings, emission reductions, and urban sanitation enhancements.

## 1 Introduction

Biological wastewater treatment (BWT) uses aeration to support a microbial community that enzymatically degrades pollutants, forming residual sludge [1]. However, traditional aeration is energy-intensive. The activated sludge process (ASP) aims to optimize aeration, reducing energy use while ensuring treatment efficacy [2]. ASP strategies include process optimization to match aeration to wastewater's oxygen needs [3], and employing efficient aeration devices like high-performance diffusers [4]. Additionally, ASP seeks to enhance pump operation and plant design for further energy savings. The ultimate goal is to meet effluent quality standards while also contributing to carbon peak and neutrality goals by adopting energy-efficient technologies [5,6].

Research in ASP seeks to balance energy efficiency with emission reduction, despite the complex, non-linear, and time-varying nature of wastewater treatment. Studies have developed models and optimization methods to improve ASP efficiency and energy savings. For instance, a novel model based on Activated Sludge Model No. 2d was calibrated using batch experiments and optimization techniques, leading to significant reductions in aeration energy and effluent quality [7]. Another study employed a multi-criteria optimization approach for an anaerobic–anoxic/nitrifying/induced crystallization (A2N-IC) process, achieving lower levels of pollutants and operation costs [8]. Further research optimized a real sewage treatment plant's performance, enhancing effluent quality and reducing energy and emissions [9]. Innovative algorithms like AMOEA/D and MOEA/D-SCS have been introduced to address complex optimization challenges in ASP, showing promise in reducing energy usage while meeting discharge standards [10,11].

ASPs must navigate the trade-offs between improving water quality, reducing energy use, and enhancing process stability [12]. Multi-criteria optimization (MCO) algorithms offer a range of Pareto solutions, providing decision-makers with various options to balance these conflicting objectives [13]. The A^2^O process, an advanced biological method, effectively removes organic substances, nitrogen, and phosphorus, and is widely implemented [[14], [15], [16]].

The goal of this research is to create a theoretical model of municipal wastewater treatment plants (WWTPs) using the improved A^2^O process. The model will be mathematically constructed, verified, adjusted, and then refined through multi-criteria optimization to improve energy effectiveness and the quality of the treated wastewater. In this study, a conventional ASM2d model was applied to simulate the real urban wastewater treatment facility, and an improved slime mould algorithm was employed to modify related parameters to enhance the precision of the model. Subsequently, the fine-tuned model was enhanced for the multi-criteria function scenario through the utilization of diverse multi-criteria improvement algorithms. This resulted in the acquisition of a group of Pareto solutions. By comparing the Pareto front curves, the most appropriate multi-criteria improvement algorithm for the study was determined. Lastly, the best approach was employed to examine the trade-off of energy conservation and emission decrease of the urban wastewater treatment facility. This research modeled a wastewater treatment system utilizing an algorithmic model and contrasted several improvement algorithms to provide experimental evidence for the energy conservation and emission decreases of wastewater treatment facilities. The examined findings guarantee a variety of decision-makers options, offering a foundation for them to select various ways for development and methods for operating.

## 2 Material and methods

### 2.1 Process methodology outline

**Fig. 1:**
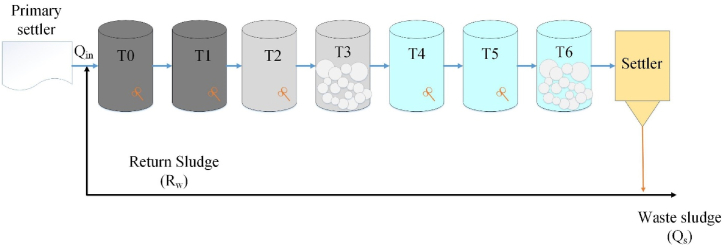
The operational framework of the enhancement the Performance of A^2^O activated sludge process.

The present study investigates the biological treatment procedure of a municipal wastewater treatment plant (WWTP) in Beijing through the utilization of a mathematical framework derived from the Activated Sludge Model No. 2d (ASM2d) and implemented in MATLAB. The municipal wastewater treatment plant employs the modified A^2^O procedure as its treatment technique. The system has eight reservoirs, each designed to fulfill distinct operational roles. The components of the system include a pre-anoxic reservoir T0, an anaerobic reservoir T1, an anoxic reservoir T2, an aerobic reservoir T3, an oxygen elimination reservoir T4, a post-anoxic reservoir T5, a post-aerobic reservoir T6, and a settler reservoir. The reservoirs for different processes had different capacities [17]. The oxygen elimination reservoir had the smallest volume of 472 m^3^, while the aerobic reservoir had the largest volume of 7461 m^3^. The other reservoirs (the pre-anoxic, anoxic, aerobic, post-aerobic, and post-anoxic reservoirs) had capacity of 1113, 1825, 5518, 945, and 1433 m^3^, respectively. The aerobic reservoir can aerate 98 m^3^ of water per minute. Fig. 1 illustrates the operational framework of the enhancement the Performance of A^2^O activated sludge process.

### 2.2 Setting model parameters and Crafting significant data

The WWTP provided the quality of the water that came in and went out, which had ${NH}_{4}^{+}$-N, COD, SS, TN, and TP. The MATLAB program made a math model that needed more parts of the water quality that came in and changed them into the 19 parts that the ASM2d model needed. The IAWQ made math models for how to treat the water with active sludge. There are four kinds of these models: ASM1, ASM2, ASM2d, and ASM3 [18]. ASM2d is employed for the concurrent removal of nitrogen and phosphorus of biological origin in activated sludge systems. Thus, this study utilized the ASM2d model for mathematical representation. Within the ASM2d model, 19 element alterations were incorporated in every bioreactor, distinguishing between dissolved and particulate forms based on their solubility, denoted as D and P, respectively [19]. The feed water is a mixture of two substances (XPAO and XAUT) that are very small in amount, so they can be ignored in this analysis. A mathematical (Takács settling) model was employed to describe how the feed water behaves in each layer [20]. The components within every level of the sedimentation reservoir model were partitioned into single particles and 9 dissolved compounds [21]. A system of differential equations was solved using the ode15s method in MATLAB.

The WWTP measured the input characteristics and amount based on common indicators such as TN, TP, ${NH}_{4}^{+}$-N, COD, SS. In order to utilize the ASM2d model, it was necessary to possess knowledge regarding the relative distribution of 19 distinct constituents within the influent organic material. Consequently, the methodology proposed by Chen et al. [7] was employed to examine the original influent water sample and derive the measurements for certain constituents. The findings are presented in Table S1. A value of 5 was assumed for the component SALK, whereas a value of 0.02 was assumed for the remaining components.

### 2.3 Calibration of the model: methods for the initial stage

A combination of mathematical optimization and sensitivity analysis are the major techniques for calibrating the primary model. Sensitivity analysis is a way of finding out how sensitive a framework is by changing the variables of the structure or the setting around it [22]. The result is more affected by alters in variables that have high sensitivity, while changes in variables that have less sensitivity have little or no effect on the result. The model calibration was done by adjusting the type variables, which were chosen from the 42 kinetic variables of ASM2d using sensitivity analysis. The sensitivity analysis was based on Equation (1), which was used to measure the sensitivity of each variable [equation (1)] [23]:

(1) $$S_{i,j}^{T}=\sum_{n=1}^{N}S_{i,j}=\sum_{n=1}^{N}\left|\frac{Y_{i,1}-Y_{i,0}}{Y_{i,0}}/\frac{P_{j,1}-P_{j,0}}{P_{j,0}}\right|$$

In the model, $S_{i,j}$ denotes the sensitivity amount of parameter i with respect to output j, where P stands for a variable parameter and Y defines a result. The values 0 and 1 represent the original and modified states, respectively, while N denotes the sample size, and $S_{i,j}^{T}$ is the total sensitivity sum. During the sensitivity study, the variable parameter was altered within a range equivalent to 10 % of its initial value.

Once the variables with higher susceptibility were identified, a mathematical improvement approach was employed to discover their precise amounts. This method revolves around converting the model's simulations and measurements into an Average Relative Deviation (ARD), whereby the kinetic variables and the ARD are treated as decision and objective variables. An optimization algorithm was utilized to minimize the average relative deviation. Equation (2) was utilized to determine the ARD [equation (2)] [24].

(2) $$ARD=\frac{1}{N}\sum_{n=1}^{N}\frac{\left|x_{i}-y_{i}\right|}{x_{i}}$$

**Fig. 2:**
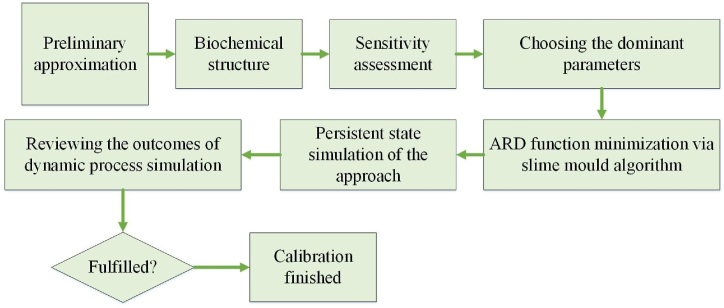
Model calibration schematic.

Fig. 2 illustrates how the kinetic parameters were calibrated. The original composition of biomass was sourced from prior research [25,26]. Then, the model was simulated, the predefined parameters were tested for their sensitivity based on the variations in the output values, and an improved slime mould algorithm was used to minimize the ARD between the observed and predicted outcomes and to obtain optimal kinetic parameters. The adjusted kinetic parameters are presented in Table S2.

### 2.4 Optimizing wastewater treatment with multiple objectives

The MCO method relies on two key elements: objective of optimization and decision variables. This study aimed to optimize three aspects: the Mean Quality of the Effluent (MQE), Total Cost Index of the process (TCI), and Overall Volume (OV). The decision variables involved the ratios of returning blended liquid and sludge ($Q_{l}$ and $Q_{s}$), the aeration efficiency coefficients in the nitrification reservoir ($K_{L}a_{1}$ and $K_{L}a_{2}$), and the volumes of the seven reservoirs in the treatment system (T1 to T7). The decision variables were used to control the process and to guide the future design and construction of wastewater treatment plants. The effluent TP, COD, TN, and ${NH}_{4}^{+}$-N also had to meet certain limits, as shown in Table S1.

In order to determine the MQE, the initial step was computing the disparity between the effluent concentration obtained from the enhanced model and the actual effluent characteristics as obtained by the wastewater treatment plant (WWTP). Subsequently, the disparity was partitioned by the factual magnitude and subsequently subjected to an averaging process [equation (3)].

(3) $$MQE=\frac{1}{n}\sum_{i=1}^{n}\frac{C_{actual}-C_{simulation}}{C_{actual}}$$

where n is the number of observations, $C_{actual}$ is the actual effluent concentration measured at the wastewater treatment plant, $C_{simulation}$ is the predicted effluent concentration.

The energy used for the operation of the system is mainly composed of the energy for pumping, aeration, and sludge treatment. The formula for calculating these energies are as below [equation (4)].

• Energy for Pumping (EP, kWhd^−1^)

(4) $$EP=\left(\int_{t0}^{t1}\left[Q_{h}\left(t\right)+Q_{w}\left(t\right)+Q_{s}\left(t\right)\right]dt\right)\times 0.04T^{-1}$$

where $Q_{h}\left(t\right)$ represents the recirculation volume of amalgamated fluid at moment t, ($m^{3}d^{-1}$); $Q_{w}\left(t\right)$ denotes the recirculation flow of sludge at the same temporal instance, ($m^{3}d^{-1}$); $Q_{s}\left(t\right)$ signifies the residual discharge of sludge at the specified time, ($m^{3}d^{-1}$). t0 is The initial time from which the pumping energy calculation starts, t1 is The final time until which the pumping energy calculation is considered, and T is The total time of the pumping process, measured in days [equation (5)].

• Energy for Aeration (EA, kWhd^−1^)

(5) $$EA=\left(\int_{t1}^{t2}{\left(Q_{in}\right)}^{-1}\sum_{i=1}^{4}\left[0.3267\times K_{L}a_{i}\left(t\right)\left(\frac{T_{i}}{T_{ref}}\right)+0.0007\times K_{L}a_{i}{\left(t\right)}^{2}\left(\frac{T_{i}}{T_{ref}}\right)\right]\right)\times 24{\left(t_{2}-t_{1}\right)}^{-1}$$

where, $Q_{in}\left(t\right)$ signifies the incoming flow rate, ($m^{3}d^{-1}$); $K_{L}a_{i}\left(t\right)$ denotes the coefficient associated with the incoming flow rate, $d^{-1}$; $T_{i}$ represents the capacity of additional biochemical reservoirs, ($m^{3}$); $T_{ref}$ is the reference temperature, typically the standard temperature at which the K_L a_i values are reported, measured in degrees Celsius (°C), t1 and t2 are the start and end times of the period over which the aeration energy is being calculated.

• Sludge generated for disposal (SG, kgSSd^−1^)

The sludge quantity within the processing mechanism at time t consists of the sludge quantity in the settling reservoir ${TSS}_{s}$ and the sludge quantity in the reaction reservoir ${TSS}_{r}$ [equations (6), (7), (8)].

(6) $$TSS\left(t\right)={TSS}_{s}\left(t\right)+{TSS}_{r}\left(t\right)$$

(7) $${TSS}_{r}\left(t\right)=\left(\sum_{j=1}^{3}\left(T_{j}\right)(Z_{I,j}+Z_{s,j}+Z_{P,j}+Z_{BH,j}+Z_{BA,j}\right))\times 0.75$$

(8) $${TSS}_{s}\left(t\right)=\left(\sum_{i=1}^{10}\left(x_{i}A\right)\left(Z_{I,i}+Z_{s,i}+Z_{P,i}+Z_{BH,i}+Z_{BA,i}\right)\right)\times 0.75$$

where, TSS(t) defines the total suspended solids in the system at time t, ${TSS}_{s}\left(t\right)$ and ${TSS}_{r}\left(t\right)$ represent the TSS in the settling reservoir and reaction reservoir in time t, respectively. $T_{j}$ is the volume of the $j^{th}$ reaction reservoir, $Z_{I,j}$, $Z_{s,j}$, $Z_{P,j}$, $Z_{BH,j}$, and $Z_{BA,j}$ denote the concentration of inert particulate material, readily biodegradable substrate, particulate products, heterotrophic biomass, and autotrophic biomass in the $j^{th}$ reaction reservoir. $x_{i}$ indicates the fraction of $i^{th}$ settling reservoir, A is the effective sectional area of the settling reservoirs, and 0.75 is a conversion factor that accounts for the density and porosity of the sludge.

The Sludge Generated (SG) encompasses the total of the gathered and released quantity of sludge in the ultimate framework, as illustrated in Equation (9):

(9) $$SG=T^{-1}\left(TSS\left(final\right)-TSS\left(first\right)+0.75\int_{t0}^{t1}\left(Z_{I,w}+Z_{s,w}+Z_{P,w}+Z_{BH,w}+Z_{BA,w}\right)Q_{w}\left(t\right)dt\right)$$

where T is the duration of the time interval in days, TSS(final) and TSS(first) define the overall suspended solids in the system at the end and beginning of the time interval. $Z_{I,w}$, $Z_{s,w}$, $Z_{P,w}$, $Z_{BH,w}$, and $Z_{BA,w}$ are the concentration of inert particulate material, readily biodegradable substrate, particulate products, heterotrophic biomass, and autotrophic biomass in the wastewater inflow, respectively. $Q_{w}\left(t\right)$ denotes the rate of flow of wastewater at a certain time t.

The calculation of the total cost index (TCI) can be performed by utilizing Equation (10):

(10) TCI=EA+EP+5SG

### 2.5 Improved slime mould optimization

The Slime Mould Algorithm (SMA) [27], which mimics how slime molds find and consume food by updating the position of individuals using an adaptive weight vector. This vector depends on each member's objective value and aims to equilibrium exploitation and exploration in the solution area. The mathematical formula of the adaptive weight vector of the SMA is given below [equation (11)]:

(11) $$\vec{W}\left(SId\left(i\right)\right)=\left\{\begin{array}{c} 1+r.\log\left(\frac{FO-C\left(i\right)}{FO-wO}+1\right),\left(A\right)\\ 1-r.\log\left(\frac{FO-K\left(i\right)}{FO-wO}+1\right),\left(B\right) \end{array}\right\}$$

where C is the collection of objective values of the individuals at every epoch which computed by the objective function, C(i) indicates the objective amount of the $i^{th}$ member. C splits the individuals into 2 groups according to the level of objective amounts, elite members with high objective amounts and mean members with low objective amounts. The first formula shows that C(i) contains the upper portion of the individuals, the second formula stands for these mean individuals, r is a random amount in the range [0,1], FO is the finest objective value achieved throughout the present iteration, wO is the worst objective value achieved throughout the present iteration. SId(i) represents the arrangement of objective values sorted.

Every member alters its location in the search area in each iteration according to Eq. (12). This approach has three major states: The initial state involves accidental search, somewhere the individual looks around the exploration area randomly with a certain chance z using uniform anytime changes. z is set to 0.03 [27]. The remaining two states imitate the foraging behavior of slime molds, wherein the organism adjusts the distance between itself and various food sources using negative and positive feedback weights, denoted as $\vec{W}$. This enables the organism to conduct a multi-point search and explore a wide range from global to local in search of the optimal solution. The equations representing these states are presented below [equation (12)]:

(12) $$\vec{y^{*}}=\left\{ \begin{array}{c} LB+\left(HB-LB\right).rand,z>rand\;\\ \vec{y_{b}\left(t\right)}+\vec{kb}.\left(W.\vec{y_{D}\left(t\right)}-\vec{y_{E}\left(t\right)}\right),p>r\\ \vec{y\left(t\right)}.\vec{kc},p\leq r \end{array}\right.$$

where LB and HB are the min and max amounts of the search interval, and r is a random number between 0 and 1. $\vec{kb}$ is defined as a variable that has a range from −a to a, $\vec{y^{*}}$ is the new location vector of slime mould particle.

The parameter a is defined as $\mathrm{arctanh}\left(1-\left(\frac{t}{-t+\max}\right)\right)$ , where t is the present iteration and −t+max is the overall number of iteration. $\vec{kc}$ reduces from 1 to 0 as the iterations increase, oscillating between [−1,1]. $\vec{y_{b}\left(t\right)}$ is the finest location, $\vec{y\left(t\right)}$ is the current location, $\vec{y_{D}\left(t\right)}$ and $\vec{y_{E}\left(t\right)}$ are two random individuals, and W is the weight of the slime mould. p stands for the likelihood strength of individuals embracing various behavioral approaches, and its formula is depicted below [equation (13)]:

(13) p=tanh|C(i)−DO|

where, i can be any number from 1 to n, C(i) is the objective of $\vec{y_{i}}$, and DO is the finest objective value achieved in all the iterations.

Improved Slime Mould Algorithm (ISMA); The SMA has demonstrated impressive results in various domains, but it may face difficulties in avoiding local optima values. This limitation leads to solutions that converge too early, even though SMA is a population-based method that has significant diversity. To address this issue, two important modifications have been proposed in this study. One approach that is used is opposition-based learning (OBL) [28]. The application of the Opposition-Based Learning (OBL) method involves the combination of both the original value and its opposite counterpart. Mathematically, the desired outcome can be obtained using the following procedure:

**Opposition-based learning:** This method is a novel technique in intelligent retrieval that has demonstrated the ability to improve several optimization approaches. OBL technique is applied to create a new opposite solution, for the problem, from the existing solution. The objective of this method is to improve the possible solution in order to approach the optimal solution by achieving a higher objective value. The opposite value of $\vec{Z}$ for the real value, where Z∈[UB,LB], is computed through equation. (14):

(14) $$\vec{y}=UB+LB-y$$

Suppose $\vec{y}=\left(y_{1},y_{2},\ldots ,y_{n}\right)$ is a point in a space with multiple dimensions, where $y_{1},y_{2},\ldots ,y_{D}\in R$ and $y_{j}\left[{UB}_{j},{LB}_{j}\right],j\in 1,2,\ldots ,D$. This representation can handle n-dimensions by using equation. (15):

(15) $${\vec{y}}_{j}={UB}_{j}+{LB}_{J}-y_{j}$$

In addition, the optimization process involves evaluating two solutions (y and $\vec{y}$) based on their objective functions, keeping the superior one, and discarding the inferior one. For the case of minimization, Y is preserved if $f\left(y\right)\leq f\left(\vec{y}\right)$; otherwise, $\vec{y}$ is preserved.

**Levy flight distribution:** Levy flight is a potent stochastic distribution technique based on non-normal distribution [29]. The position of the solution is updated using the Levy flight procedure according to equations (16), (17):

(16) $$\vec{y\left(t+1\right)}=\vec{y_{b}\left(t\right)}\times Levy\left(\vec{D}\right)$$

(17) $$Levy\left(\vec{D}\right)=\frac{\sigma \times u}{{\left|\upsilon \right|}^{1/\beta }}\times s$$

The value of s is a fixed constant of 0.01, u and υ are random amounts from 0 to 1. υ is computed as follows [equation (18)]:

(18) $$\upsilon =\left(\frac{sine\left(\frac{\pi \beta }{2}\right)\times \Gamma \left(\Gamma +1\right)}{\beta \times 2^{\left(\beta -1/2\right)}\times \Gamma \left(1+\beta /2\right)}\right)$$

where sine defines the value of the sine function, and β is equal to 1.5.

Therefore, the algorithm has three parts: SMA, OBL, and Levy flight. It randomly creates solutions and tests two conditions to pick apart. The first condition is rand<0.9. If yes, it does SMA. If no, it tests the second condition: rand≤0.5. If yes, it does OBL. If not, it does Levy flight. The algorithm does these steps: set parameters, create solutions, calculate fitness value, find best and worst, find W and a, test conditions, pick part, and check finish.

#### 2.5.1 CRFSMA

SMA is an algorithm that uses two methods to keep variability: local and global exploration. Global exploration lets individuals pick randomly with uniform values in the decision area. Local exploration uses adaptive $\vec{W}$ to search at many points at once. But SMA's global search is not ergodic and may miss diverse solutions in complex spaces. To fix this and improve variability in intricate multi-criteria cases, two methods were suggested [30]. First, chaos was used to make SMA travel better in the decision area. With chaos, SMA can explore further region faster and find more diverse solutions. Second, as the objectives grow, keeping diversity is harder. To solve this, the Pareto-based non-dominated sorting method is used [31] to keep diversity. Through combining these two methods with SMA a novel method called CRFSMA is developed [30]. This method uses the benefits of chaotic travel and diversity based on reference points to make SMA better at (addressing intricate multi-criteria challenges.

⁃ One-Dimensional Logistic Chaos Perturbation for Global Search

The phenomenon of chaos, characterized by a state of unpredictability and disorder, occurs in systems of non-linear dynamics and is often seen in nature and society. The logistic map [32] is a well-known dynamical system of one-dimensional chaotic. It has been extensively employed to augment the explore prowess of evolutionary strategies across various domains. The major idea behind logistic chaotic single-dimension perturbation involves utilizing pseudo-random amounts produced via logistic chaotic mappings [33]. This method suggests numerous benefits. The chaotic sequence's ergodicity increases the perturbation result's unpredictability, resulting in a broader spread in the search space. Also, perturbing only one dimension maintains the present best solution's desirable attributes and prevents inefficient searches resulting from large-scale irregular perturbations. The logistic chaotic single-dimension perturbation approach perturbs each individual through logistic mapping of a one-dimensional, which is represented by formula 14 [equation (19)].

(19) $$y_{i}={lb}_{i}+M\left(t\right)\left({ub}_{i}-{lb}_{i}\right)$$

where, ${lb}_{i}$ and ${ub}_{i}$ reflect the lower and upper limit of the decision variable for the $i^{th}$ dimension. The function M(t) denotes a pseudo-random number that is produced by a logical chaotic system. These pseudo-random numbers are influenced by time and adhere to the laws of chaotic dynamics. Chaos is the term used to describe a type of movement that is not repeated and begins from a specific beginning point. This movement generates a random sequence that shows a higher level of ergodicity than a uniform distribution. Equation (15) provides the mathematical depiction of chaos [equation (20)]:

(20) M(t+1)=(1−M(t)).μM(t)

where, M(t) represents the $t^{th}$ chaotic sequence (where 1≤t≤(n−1)). The parameter μ ranges between 1 and 4, with a value closer to 4 resulting in a more ergodic chaotic sequence. Therefore, the mechanism sets μ to 4. Furthermore, chaos is specifically that M(t) does not equal 0.25, 0.5, 0.75, or 1.

⁃ Reference points for non-dominated sorting

Selection is vital and different in multi-criteria optimization, which has many non-dominated solutions as the number of objectives grows. In multi-criteria optimization, a ranking method is needed to choose a Pareto solution group that is diverse and convergent. The basic SMA has the benefit of multi-point explore, which helps the variety of good solutions. To use this benefit in multi-criteria cases, a method based on non-dominated sorting and reference points was proposed in 2013 to choose a Pareto solution set that has variety and convergence for the next generations [34].

The non-dominated sorting approach, which relies on reference points, encompasses two primary processes: non-dominated sorting and reference point identification. Non-dominated sorting utilizes the conception of Pareto dominance association to evaluate and choose the Pareto solution set. It takes a solution set Y as input and produces a set F that contains several rank sets ($F_{1},F_{2},\ldots ,F_{l}$). Each rank set $F_{i}$ represents the group of non-dominated solutions at the $i^{th}$ level of rank. The amount of rank levels is denoted by l. The specific steps are explained below, with theoretical proof in Ref. [35].

⁃ Give each solution p two values: np (how many solutions are better than p) and Sp (which solutions are worse than p).
⁃ Start with i=1 and put solutions with np=0 in the first group $F_{i}$.
⁃ For each solution p in $F_{i}$, lower nq for each solution q in Sp. If nq becomes zero, move q to the next group $F_{i+1}$.
⁃ The input solution set Y is sorted into groups by Pareto dominance. The finest solutions that are not dominated by any other are in $F_{1}$. If $F_{1}$ has too few solutions, apply the reference point technique from Ref. [35] to enhance the algorithm.

**Fig. 3:**
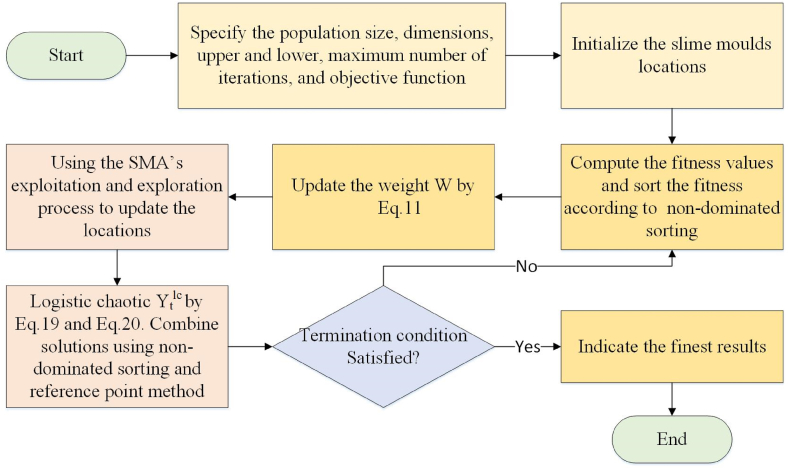
The structured methodology of CRFSMA.

To complement the non-dominated sorting method, the method of reference point is used. It selects T individuals from $F_{l}$ to fill the population size N, when the sum of elements in $F_{1},F_{2},\ldots ,F_{1-l}$ is less than N but greater than the sum of elements in $F_{1},F_{2},\ldots ,F_{l}$ (the number of elements in $F_{l}$ is $\left|F_{l}\;\right|$). The processes contain 4 parts: 1) The objective area is normalized to make the optimization results comparable. 2) The reference point vector $Z^{s}$ is set by using the uniform point function in MATLAB, which generates points that are evenly spaced on the unit hyperplane. 3) Each individual is linked to a reference point and the number of members for every reference point is calculated. 4) T members from $F_{l}$ are chosen according to the link data. Fig. 3 presents the structured methodology of CRFSMA.

### 2.6 Algorithm validation

Test functions play a crucial role in practical mathematics as they allow for the evaluation of optimization algorithms based on their variety, convergence accuracy, and robustness. A total of 10 benchmark functions have been selected for this study. Each function is described in detail in Table 1, which includes the size of the decision space (D), the number of objectives (M), the bounds of the decision space, and the maximum allowable number of iterations for each function. Four novel multi-criteria evolutionary algorithms are carefully chosen for the purpose of comparison. These algorithms include NSGA-III (Non-dominated Sorting Genetic Algorithm III) [36], IBEA (Indicator-Based Evolutionary Algorithm) [37], MOEA/D-RA (Multi-criteria Evolutionary Algorithm Based on Decomposition with Reference-point Adaptation) [38], MOPSO (Multi-criteria Particle Swarm Optimization) [39]. The proposed CRFSMA's performance was evaluated using the Inverted Generational Distance (IGD) [40]. The IGD is a thorough measure for assessing performance, comparing the excellence of the real Pareto front and the acquired approximate solution. The algorithm's convergence and dispersion can be assessed by determining the minimal total distances of Euclidean between each point (member) on the genuine PF and the solution set generated by the algorithm. Assuming Q represents the non-dominant solution set created by a multi-criteria algorithm, and P denotes the reference point set uniformly sampled along the authentic PF, the IGD amount can be computed in the following manner [equation (21)]:

(21) $$IGD\left(P,Q\right)=\frac{\sum_{\nu \in p}\min_{X\in Q}{\left\|\nu ,X\right\|}_{2}}{\left|P\right|}$$

where, |P| indicates the magnitude of set P, and ${\left\|.\right\|}_{2}$ symbolizes the distance of Euclidean. If the average IGD amount of the test function is lower, this indicates a superior algorithm.

**Table 1:** Specification of test functions.

Functions	D	M	Bounds	Max iteration
UF1	30	2	[-1, 1]	1000
UF2	30	2	[-1, 1]	1000
UF3	30	2	[-1, 1]	1000
ZDT1	30	2	[0, 1]	1000
ZDT2	30	2	x1ϵ [0, 1], xiϵ [-5, 5]	1000
ZDT3	30	2	[0, 1]	1000
DTLZ1	10	3	[0, 1]	1000
DTLZ2	10	3	[0, 1]	1000
DTLZ3	10	3	[0, 1]	1000
DTLZ7	10	3	[0, 1]	1000

To ensure a fair evaluation of any method, this analysis was repeated thirty times. Table 2 shows the IGD metric for each test function. The IGD metric indicates the averaged IGD values of every algorithm after 20 independent runs on every function, indicated as StD and Avg values.

**Table 2:** Comparison of IGD results values on 10 test functions.

Function	Indicator	NSGA-III [36]	IBEA [37]	MOEA/D-RA [38]	MOPSO [39]	CRFSMA
F1	Avg	8.745E-02	2.267E-01	1.203E-01	9.041E-02	**3.52E-02**
	StD	1.700E-02	1.610E-02	7.070E-02	2.100E-02	1.11E-02
	Min	7.045E-02	2.106E-01	4.961E-02	6.941E-02	2.41E-02
	Max	1.0445E-01	2.428E-01	1.710E-01	1.114E-01	4.63E-02
F2	Avg	3.040E-02	1.231E-01	1.37E-02	3.102E-02	**4.100E-022**
	StD	6.611E-03	3.720E-02	1.46E-03	1.120E-02	2.140E-02
	Min	2.378E-02	8.59E-02	1.224E-02	1.98E-02	1.96E-02
	Max	3.701E-02	1.603E-01	1.516E-02	4.224E-02	6.24E-02
F3	Avg	2.232E-01	1.053E-01	3.054E-01	1.765E-01	**8.62E-02**
	StD	5.240E-02	3.010E-02	3.820E-02	5.210E-02	8.71E-03
	Min	1.708E-01	7.52E-02	2.672E-01	1.244E-01	7.749E-02
	Max	2.756E-01	1.354E-01	3.436E-01	2.286E-01	9.491E-02
F4	Avg	3.776E-03	7.303E-02	4.560E-03	3.878E-03	**3.52E-03**
	StD	6.721E-07	1.182E-02	2.531E-04	8.240E-05	8.01E-05
	Min	3.775E-03	6.121E-02	4.307E-03	3.870E-03	3.439E-03
	Max	3.777E-03	8.485E-03	4.813E-03	3.886E-03	3.601E-03
F5	Avg	3.616E-03	1.297E-01	5.317E-03	3.742E-03	**3.41E-03**
	StD	8.251E-08	2.342E-02	8.160E-04	5.132E-05	9.13E-05
	Min	3.615E-03	1.073E-01	4.501E-03	3.691E-03	3.319E-03
	Max	3.617E-03	1.521E-01	6.133E-03	3.793E-03	3.501E-03
F6	Avg	6.016E-03	7.416E-2	1.926E-02	5.768E-03	**5.42E-03**
	StD	1.735E-04	1.410E-02	1.180E-02	5.240E-03	7.15E-04
	Min	5.843E-03	6.006E-02	7.460E-03	5.244E-04	4.705E-03
	Max	6.189E-03	8.826E-02	3.106E-02	1.129E-02	6.135E-03
F7	Avg	2.167E-02	2.570E+01	2.391E-02	2.121E-02	**8.18E-03**
	StD	2.281E-06	4.060E+00	7.810E-03	1.180E-04	6.22E-04
	Min	2.164E-02	2.164E+01	1.610E-02	2.119E-02	7.558E-03
	Max	2.170E-02	2.976E+01	3.172E-02	2.123E-02	8.802E-03
F8	Avg	5.340E-02	3.835E-01	5.345E-02	5.205E-02	**1.54E-03**
	StD	1.321E-04	4.071E-01	5.210E-06	4.871E-04	1.27E-04
	Min	5.328E-02	−2.360E-02	5.340E-02	5.156E-02	1.413E-03
	Max	5.352E-02	7.792E-01	5.350E-02	5.254E-02	1.66E-03
F9	Avg	5.558E-02	1.778E+02	5.559E-02	5.431E-02	**5.821E-02**
	StD	9.640E-06	1.841E+01	2.160E-05	6.881E-04	3.57E-02
	Min	5.548E-02	1.594E+02	5.557E-02	5.362E-02	2.251E-02
	Max	5.568E-02	1.962E+02	5.561E-02	5.500E-02	9.391E-02
F10	Avg	9.670E-02	2.563E-01	1.874E-01	6.859E-02	**7.13E-02**
	Min	2.359E-02	9.52E-02	3.43E-02	1.739E-02	6.644E-02
	Max	1.698E-01	4.173E-01	3.405E-01	1.198E-01	7.616E-02
	StD	7.310E-02	1.610E-01	1.531E-01	5.120E-02	4.856E-03

The algorithm performance is better when the IGD value is smaller. CRFSMA has smaller average IGD values for test functions as shown in Table 2. This means that CRFSMA can consistently find optimal solutions for these functions.

## 3 Findings and analysis

### 3.1 Analysis of wastewater composition and model performance

**Fig. 4:**
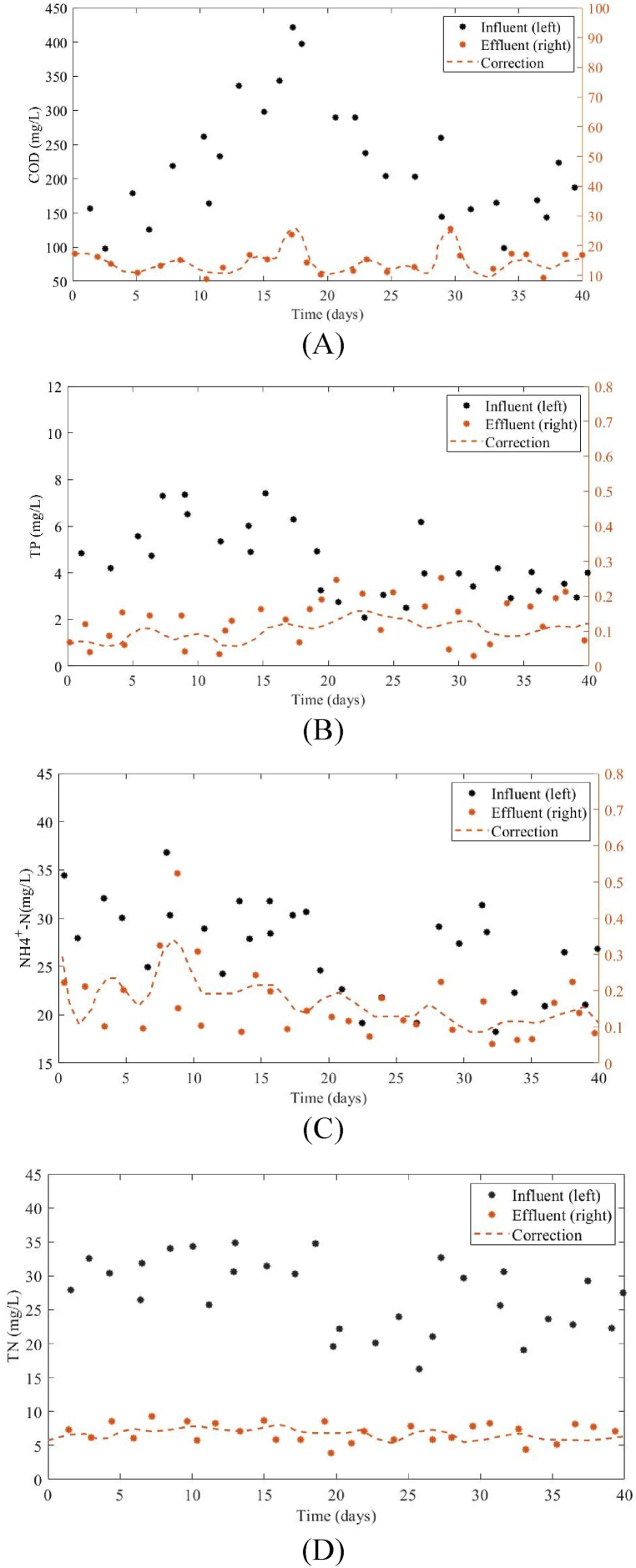
The variation of influent quality in terms of the four components including (A) COD, (B) TP, (C) ${NH}_{4}^{+}$-N, and (D) TN during a 40-day dynamic influent scenario, and the difference among the observed and adjusted model outcomes.

The COD of the influent from real WWTPs varied greatly, from 98 to 422 mg/L. This variability can be attributed to the combined impact of precipitation and sewage flow during the period of high precipitation, as illustrated in Fig. 4. The TP, ${\text{NH}}_{4}^{+}$-N, and TN of the influent also varied greatly, with STD amounts of 5.83, 5.99, and 1.87 mg/L. This was due to the coexistence of companies in the vicinity, where manufacturing effluents were combined with the sewage system.

In the analysis of wastewater composition and model performance, Total Nitrogen (TN) is a comprehensive indicator that includes all forms of nitrogen compounds present in the wastewater. As depicted in Fig. 4, the TN concentration in the influent is higher than the Ammonium Nitrogen (${NH}_{4}^{+}$-N) levels. This is because TN encompasses (${NH}_{4}^{+}$-N) along with other nitrogen species. The variability in TN and (${NH}_{4}^{+}$-N) concentrations reflects the complex nature of wastewater, which can be influenced by industrial effluents and other sources. Similarly, in the effluent, the TN content remains higher than (${NH}_{4}^{+}$-N) because (${NH}_{4}^{+}$-N) is a subset of TN. The model's ability to predict these values accurately is crucial for assessing the efficiency of the wastewater treatment process. In Fig. 4. C, the (${NH}_{4}^{+}$-N) values in the effluent state correspond to the Y-axis on the right side, while the (${NH}_{4}^{+}$-N) values in the influent state correspond to the Y-axis on the left side. The axes are marked with two different colors to differentiate them clearly, aiding in the visual interpretation of the data.

The utilization of computational models for quantifying the parameters of wastewater treatment plants gives rise to discrepancies among the observed and modeled results. The errors seen in this study are deemed acceptable since they exhibit a level of comparability to discrepancies resulting from the variation in daily wastewater inflow, precipitation patterns, and the irregular level of phosphorus and nitrogen. Using the process of model fine-tuning, the discrepancy amount is minimized in a predetermined boundary, hence guaranteeing the congruence between the modeled data and the corresponding observed values. This research concentrated on the multi-objective optimization technique to optimize the impact of the effluent characteristics with the restricted calibration discrepancies. To validate the accuracy of the simulation, the outcomes of the modified sensitivity were compared with the effluent and influent data from the original sewage plant (Fig. 4). The simulation's water quality discrepancy, upon verification, fell within an acceptable range. This indicates that the model was able to accurately mimic the actual features and satisfy the demands of the research.

The existing model has the capability to accurately replicate the wastewater process according to the level of conformity between the scatter and curve. The total influent quality of WWTP varied significantly. The total variations in water quality, as determined through simulation and measurement, were alike and did not surpass the accepted benchmarks for wastewater quality. Specifically, the absolute relative differences (ARD) for TN, COD, TP, and ${NH}_{4}^{+}$-N were calculated to be 25.32 %, 14.17 %, 28.21 %, and 47.35 % (Fig. 4). The mean amounts for TN, COD, TP, and ${\text{NH}}_{4}^{+}$-N were 5.98, 14.43, 0.12 and 0.18 mg/L. Similarly, the mean modeled amounts for these parameters were found to be 5.03, 12.05, 0.08 and 0.04 mg/L, indicating that the model can reliably simulate the wastewater treatment process. The mean values for these parameters, both observed and modeled, further support the model's capability to replicate real-world conditions. In conclusion, the study demonstrates that despite the challenges posed by influent variability and external industrial influences, computational models, when properly calibrated and optimized, can effectively predict wastewater treatment outcomes.

### 3.2 Analysis and set of optimal solutions based on Pareto criterion

**Fig. 5:**
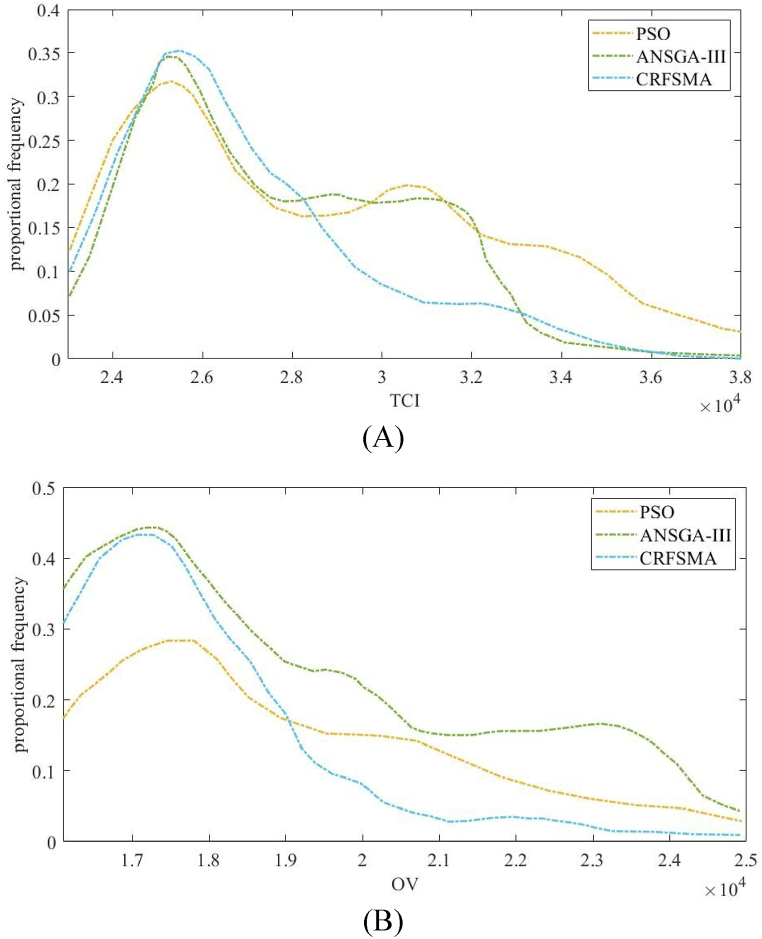
Evaluating different MCO algorithms for biological wastewater treatment optimization over 40 days: (A) PSO and ANSGA-III and (B) CRFSMA convergence at an alternative point, different from the highest peak.

A way to evaluate the progress of the optimization process is to use the distribution of all (solution) sets for the final iteration. The Pareto optimal set surfaces also provide a clearer indication of the suitability of the optimization algorithm. The majority of solution groups obtained from the three optimization algorithms have a range between 25000 kWh⋅d^−^^1^ and 17,000 m^3^. The process of convergence in different algorithms is shown in Fig. 4. The presence of peaks indicates that all three algorithms have achieved convergence. In Fig. 5 (A) PSO and ANSGA-III methods have converged at an alternative point, different from the highest peak. Similarly, in Fig. 5 (B), the CRFSMA method also demonstrates a visible low peak, indicating convergence at a different location. The general pattern was consistent for all processes, and the CRFSMA process achieved a solution set that was both faster and more accurate than the other two methods.

**Fig. 6:**
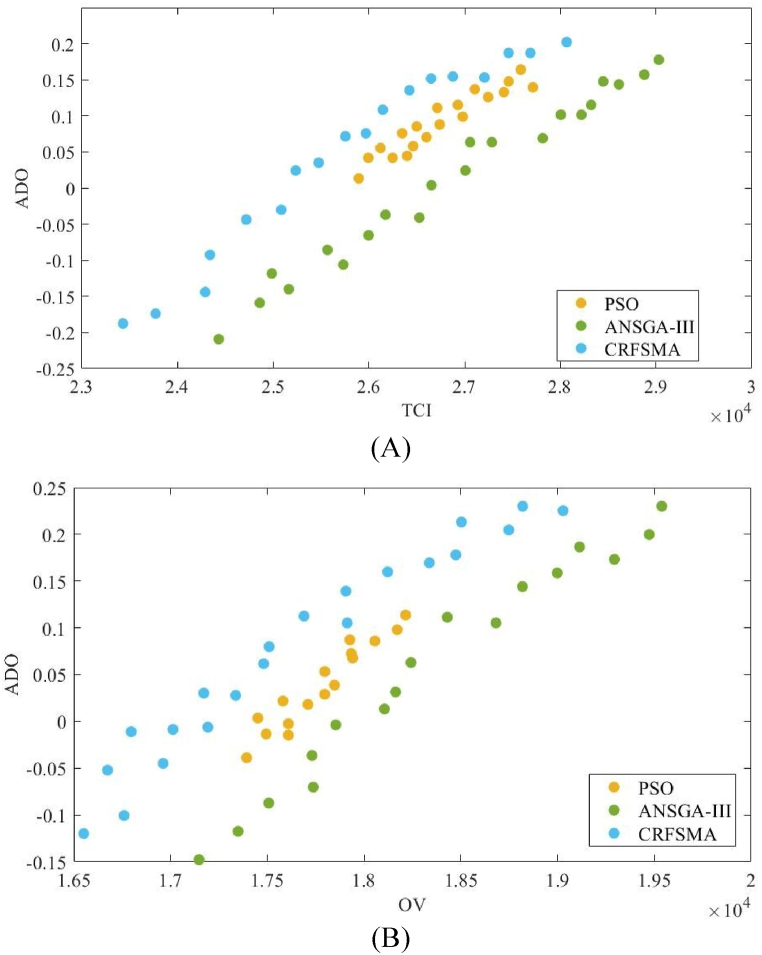
Energy consumption and overall volume histograms and boxplots from the final epoch of MCO for (A) TCI, and (B) OV.

Two groups of objectives are chosen from three groups, taking into account the spatial variance of the three-dimensional graph, in order to generate a clear two-dimensional graph. Fig. 6 present two-dimensional charts illustrating the mean levels of water quality improvement, energy utilization, and capacity improvement outcomes among the three various methods. The charts often exhibit negative values for average improvement metrics, which is deemed acceptable in this work. The objective of this approach is to increase the opportunities for the enhancement algorithm, hence removing any limitations on the provision of a simulated output quality that may surpass the actual output quality. Hence, it is imperative to exclude it from the range of optimization methodologies.

As illustrated in Fig. (6), the particle swarm method's Pareto front surface approaches the optimal region in a uniform manner. The entire Pareto front surface changes based on the evolution of the global finest solution. In contrast to the population of ANSGA-III and CRFSMA, the particle swarm algorithm reaches the optimal region more quickly, but it is also more susceptible to getting stuck in a local optimum, leading to a worse set of optimal solutions. ANSGA-III converges more slowly than PSO because of its high computational cost and lack of elitism, while CRFSMA converge faster than PSO because of its adaptive reference point selection and crowding distance measurement. In addition, both ANSGA-III and CRFSMA have higher quality solutions than PSO because they can search different areas of the search space and prevent early convergence. As Fig. 6 show, the Pareto front curves of ANSGA-III and CRFSMA had alike patterns. The Pareto front curve of CRFSMA was superior to that of ANSGA-III, meaning that CRFSMA had better average quality solutions than ANSGA-III. Thus, the CRFSMA algorithm was more appropriate for high-dimensional optimization problems.

To extract a solution from the Pareto front, specifically for this study on the optimization of biological wastewater treatment, the following can be mentioned:

Identify the Pareto Optimal Set: We examine the solution sets from the final iteration of multi-criteria optimization (MCO) to identify the non-dominated set. This set represents the best trade-offs between energy consumption, overall volume, and mean quality of effluent (MQE).

Assess Convergence and Distribution: By observing the convergence patterns in the Pareto optimal set, we can assess the reliability of the solutions. In our study, the CRFSMA method showed a faster and more accurate convergence, suggesting its solutions may be preferable.

Choose Objectives for Comparison: From the three-dimensional optimization graph, we select two objectives to generate a clear two-dimensional graph for comparison.

Analyze the Two-Dimensional Graphs: These graphs illustrate the mean levels of improvement in water quality, energy utilization, and capacity outcomes. They guide us in selecting a solution that best meets our criteria for the urban sanitation facility.

Evaluate Algorithm Performance: The performance of the CRFSMA algorithm, indicated by its Pareto front surface and convergence pattern, influences our choice. Its superior average quality solutions make it a more suitable choice for our high-dimensional optimization problem.

Select a Solution: Based on this analysis, we would choose a solution from the CRFSMA algorithm's Pareto front curve. This solution effectively balances the trade-offs between our objectives while optimizing the 12 process parameters.

By following these steps, we can extract a solution that not only meets our performance measures but also contributes to significant improvements in wastewater quality and reductions in energy utilization, as demonstrated by the quantitative improvements in our study.

### 3.3 Evaluating the optimization and main strategies

**Fig. 7:**
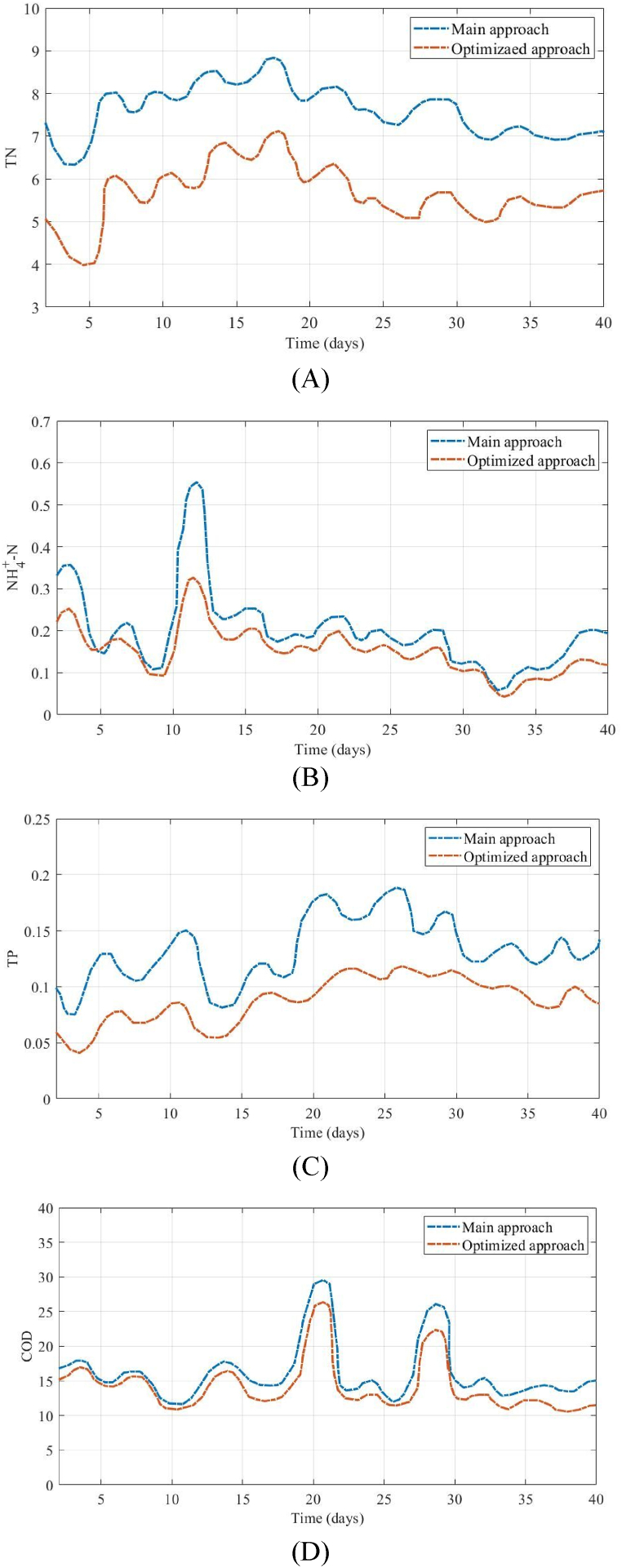
Effect of optimization strategy on the quality of wastewater under dynamic influents for (A) TN, (B) ${NH}_{4}^{+}$-N, (C) TP and (D) COD.

Considering the small disparity in the mean level of treated wastewater improvement, the selected subset with the lower energy utilization and overall volume of the reactor was selected from the CRFSMA optimal solution group. Energy utilization and capacity in the optimized procedure reduced from 25,831 kWh⋅d^−^^1^ and 18,107 m^3^ under the default procedure to 24,396 kWh⋅d^− 1^ and 17,283 m^3^, respectively. The decrease in the volume of the biochemical reservoirs was mainly in the post-aeration reservoirs, aerobic, and anaerobic; they went down from 945 m^3^, 7461 m^3^ and 1825 m^3^, to 838.5 m^3^, 7031.2 m^3^ and 159.5 m^3^. The mixture's internal recirculation ratio rose a bit, from 320 % to 390 %, leading to an elevated return flow rate. At the same time, the rate of sludge removal went down from 100 % to 88 % and the nitrification reservoir's oxygen transfer rate went up from 248 to 269. The improvements implemented led to an enhance in the oxygen quantity and staying duration within the system, for water and sludge. This enhancement contributed to the better removal of nitrogen and phosphorus. Fig. 7 illustrates a comparative analysis of the water quality achieved through both the main and optimized approaches under a dynamic inflow situation spanning a duration of 40 days. Overall, there was a 14.8 % improvement in the quality of the effluent.

**Fig. 8:**
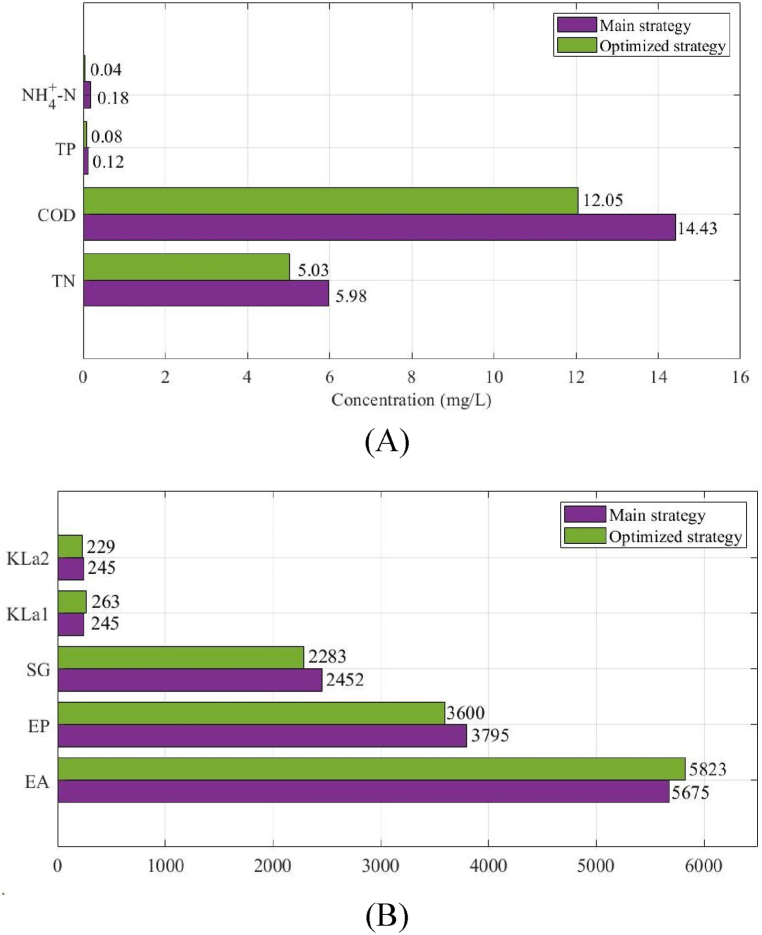
Effect of wastewater quality and energy efficiency between optimized and main strategies over 40-day influent circumstances: (A) comparison of the mean concentrations of TN, COD, TP and ${NH}_{4}^{+}$-N in the enhanced effluent, (B) The observed enhancement in the coefficient of oxygen transfer.

The graph illustrates that there is a trend towards achieving improved overall water quality, while there are concerns with the TP and ${NH}_{4}^{+}$-N outputs. This phenomenon may be attributed to the lower amounts of TP and ${NH}_{4}^{+}$-N in the effluent, which exhibit variability at lower levels as a result of the inflow volume's influence. The mean concentrations of the effluent which optimized exhibited a decrease in comparison to the initial approach. Fig. 8 (A) presents a comparison of the mean concentrations of TN, COD, TP and ${NH}_{4}^{+}$-N in the enhanced effluent. These concentrations exhibited a decrease of 0.95, 2.38, 0.04 and 0.14 mg/L, respectively. The observed enhancement in the coefficient of oxygen transfer likely resulted in a higher aeration consumption and an enhancement in use of aeration energy from 5675 kWh⋅d^−^^1^ to 5823 kWh⋅d^− 1^ in the energy use in Fig. 8 (B). The utilization of energy by the pump and the discharge of sludge were both seen to decrease by 5.12 % and 6.89 %, respectively. In summary, the energy usage in the enhanced approach was shown to be reduced compared to the main approach.

To better compare optimization algorithms in this study and also to show the effectiveness of processing optimization algorithms in WWTP, Table 3 is provided.

**Table 3:** Comparative analysis of optimization algorithms in wastewater treatment.

Metrics	Algorithm		
	CRFSMA	PSO	ANSGA-III
Energy consumption reduction rate	18.83 %	13.63 %	15.96 %
OV reduction rate	18.27 %	16.24 %	15.96 %
MQE increase rate	14.8 %	10.68 %	12.35 %
TCI reduction (mg/L)	7.4 %	5.95 %	6.37 %
TN reduction(mg/L)	0.95	0.83	0.89
COD reduction (mg/L)	2.38	1.97	2.25
${NH}_{4}^{+}$-N reduction (mg/L)	0.04	0.02	0.026
TP reduction (mg/L)	0.14	0.11	0.12

As is clear from the table, CRFSMA consistently outperforms the other two algorithms across all metrics, indicating its effectiveness in optimizing WWTP operations. It not only reduces energy consumption and overall volume significantly but also improves the quality of effluent while reducing the concentration of various contaminants. This suggests that CRFSMA could be a more suitable choice for achieving enhanced performance in biological wastewater treatment processes.

## 4 Conclusions

This research developed an adapted A^2^O framework based on real WWTPs by applying mathematical modeling techniques. The framework was validated through the analysis of sensitivity and improved slime mould method, and the projected outcomes matched well with the observed effluent variables from the WWTP. Distinct MCO techniques were employed for simulating and optimizing the energy consumption, overall volume, and MQE of the approach. The three optimization techniques were evaluated by their optimal trade-offs and Pareto frontier, and the results showed that the CRFSMA performed finer for many-dimensional objectives. The optimized approach enhanced the overall effluent quality by 14.8 % compared to the common approach, and the improved effluent had a lower percentage of TN, COD, TP and ${NH}_{4}^{+}$-N by 0.95, 2.38, 0.04 and 0.14 mg/L. Concurrently, the overall volume and energy utilization were lowered by 18.27 % and 18.83 %, respectively. The optimization of ASPs is a challenging problem that requires high efficiency and accuracy. ASPs are systems that have properties that change over time and depend on each other. Therefore, simple optimization algorithms may not be able to capture the complexity and dynamics of ASPs. Future research should look into more complex optimization algorithms that can handle the time-varying and coupled nature of ASPs. These algorithms may be able to find better solutions for optimizing ASPs than the existing ones.

## Data availability statement

Research data are not shared.

## CRediT authorship contribution statement

**Chunchang Lu:** Formal analysis, Data curation, Conceptualization. **Ying Chen:** Formal analysis, Data curation, Conceptualization. **Behrooz Eskandarpour:** Formal analysis, Data curation, Conceptualization. **Khalid A. Alnowibet:** Writing – review & editing, Validation, Formal analysis, Data curation, Conceptualization.

## Declaration of competing interest

The authors declare that they have no known competing financial interests or personal relationships that could have appeared to influence the work reported in this paper.
