# Supplementary material for: Optimization of a real municipal sewage treatment plant using CRFSMA algorithm and a mathematical model
Source: Heliyon. 2024 Jul 21;10(15):e34785. doi: 10.1016/j.heliyon.2024.e34785 (PMC11336287; doi:10.1016/j.heliyon.2024.e34785)
Supplement: Multimedia component 1 [file mmc1.docx]

Table S1. The properties and composition of the influent

| **Symbol** | **Unit** | **Ranges** |
| --- | --- | --- |
| BCOD | mg COD/L | [142.41, 197.51] |
| ${COD}_{f}$ | mg COD/L | [74.94, 104.72] |
| ${COD}_{inf}$ | mg COD/L | [201.95, 282.19] |
| TSS | mg SS/L | [117.96, 157.76] |
| TP | mg P/L | [27.51, 38.23] |
| $\mathrm{BOD}_{5}$ | mg COD/L | [90.58, 125.62] |
| TN | mg N/L | [27.51, 38.23] |
| NO_3_-N | mg N/L | [0.32, 1.22] |
| $\mathrm{PO}_{4}^{3-}$-P | mg P/L | [1.73, 2.67] |
| $\mathrm{NH}_{4}^{+}$-N | mg N/L | [21.09, 29.13] |
| $f_{BOD}$ | $-$ | 0.67 |
| $f_{XI}$ | $-$ | 0.75 |
| $Y_{H}$ | mg / mg COD | 0.676 |

Table S2. Comparison of initial and calibrated amounts for sensitive dynamic parameters

| **Explanation of parameters** | **Intervals** | **Initial amounts** | **Calibrated amounts** |
| --- | --- | --- | --- |
| **Hydrolysis** |  |  |  |
| Rate coefficient for hydrolysis | Between 0.96 and 4.5 | 3.5 | 4.862 |
| Diminishing factor for hydrolysis under anoxic conditions | Between 0.55 and 0.9 | 0.63 | 0.896 |
| **Heterotrophic** |  |  |  |
| Highest increase rate of XH | Between 0.6 and 13.2 | 6.95 | 5.85 |
| Bacterial lysis kinetics | Between 0.05 and 1.6 | 0.5 | 0.714 |
| **PAO** |  |  |  |
| Constant rhythm for XPHA storage | Between 2 and 6 | 4 | 2.157 |
| Maximum fraction of XPP over XPAO | [Between 0.2 and 0.51 | 0.36 | 0.498 |
| Constant rhythm for XPP storage | Between 1 and 1.5 | 1.4 | 1.479 |
| Modulator for anoxic activity | Between 0.55 and 0.9 | 0.61 | 0.9 |
| **Nitrobacteria** |  |  |  |
| Highest rate of increase rate of XAUT | Between 0.2 and 1.2 | 1 | 1.198 |
| Equilibrium constant of nitrogenous ammonia | Between 0.1 and 2 | 1.4 | 1.2 |
